# Gunning on Jaw Fractures

**Published:** 1880-12

**Authors:** James E. Garrettson

**Affiliations:** No. 1537 Chestnut st., Philadelphia


ARTICLE II.

### GUNNING ON JAW FRACTURES.

BY PROF. JAMES E. GARRETTSON, M. D., OF PHILADELPHIA.

MESSRS. EDITORS.—Will you kindly allow me the space of a paragraph in which to frame a small picture?

A wand of wood, bent arch fashion, rests by its extremities upon a table. Intermediate to these supports there is found a staple driven into the boards. From this staple there runs two elastic bands. The first of these bands is firmly attached to the centre of the arch. The second of the bands loses itself in the first about midway between staple and arch. Both pull on the arch.

The paragraph is the picture. The picture is the diagram of the genii-hyoid and the hyo-glossus muscles to each other, to the hyoid bone and to the chin part of the lower jaw bone.

Oblige me by asking any school boy you may happen to number among your acquaintances what relation to the staple would be assumed by that portion of the wand-arch to which the elastic band is attached should accident dissever the portion?

When it shall have happened Thomas Brian Gunning, D. D. S., to have treated a sufficient number of fractures to have possessed him of a desirable experience, he will know that muscles, like elastic bands, displace in the direction of their contractility; no antagonism to the displacement existing.

Reference is not anywhere made in "A Treatise on Diseases and Surgery of the mouth, jaws, etc." to any "bandage" in connection with "my splints;" There is, however, a reference which reads thus: In case of displacement, Dr. Gunning uses an appliance similar to one previously suggesting itself to Mr. Nasmyth, not so good or convenient as that by the English surgeon, as it unnecessarily covers too many teeth and thus is made cumbersome.

The gentleman having assumed the part of a critic, it will not be amiss to tell him that reference to his work, in the volume he refers to arose out of a desire to encourage him in the efforts he seemed making; not certainly from any admiration of his apparatus. The perfection of surgery is simplicity. Dr. Gunning's splints are the perfection of obscurity. A skillful and experienced practitioner requires seldom more than a piece of pasteboard and a strand of wire to cure any case of jaw fracture.

No. 1537 Chestnut st., Philadelphia.

